# Unveiling the Complexity of Red Clover (*Trifolium pratense* L.) Transcriptome and Transcriptional Regulation of Isoflavonoid Biosynthesis Using Integrated Long- and Short-Read RNAseq

**DOI:** 10.3390/ijms222312625

**Published:** 2021-11-23

**Authors:** Kun Shi, Xiqiang Liu, Xinyi Pan, Jia Liu, Wenlong Gong, Pan Gong, Mingshu Cao, Shangang Jia, Zan Wang

**Affiliations:** 1 College of Grassland Science and Technology, China Agricultural University, Beijing 100193, China; 15600912620@163.com (K.S.); xiqiangliu003@126.com (X.L.); Liujia199301@163.com (J.L.); shangang.jia@cau.edu.cn (S.J.); 2 Institute of Animal Sciences, Chinese Academy of Agricultural Sciences, Beijing 100193, China; Panxinyi19@163.com; 3 Pratacultural College, Gansu Agricultural University, Lanzhou 730070, China; gongwl2019@163.com; 4 Institute of Plant Protection, Chinese Academy of Agricultural Sciences, Beijing 100193, China; gp68x@126.com; 5 Grasslands Research Centre, AgResearch Limited, Palmerston North 4410, New Zealand; Mingshu.Cao@agresearch.co.nz

**Keywords:** red clover, transcriptome, isoflavonoid biosynthesis, transcription factor

## Abstract

Red clover (*Trifolium pratense* L.) is used as forage and contains a high level of isoflavonoids. Although isoflavonoids in red clover were discovered a long time ago, the transcriptional regulation of isoflavonoid biosynthesis is virtually unknown because of the lack of accurate and comprehensive characterization of the transcriptome. Here, we used a combination of long-read (PacBio Iso-Seq) and short-read (Illumina) RNAseq sequencing to develop a more comprehensive full-length transcriptome in four tissues (root, stem, leaf, and flower) and to identify transcription factors possibly involved in isoflavonoid biosynthesis in red clover. Overall, we obtained 50,922 isoforms, including 19,860 known genes and 2817 novel isoforms based on the annotation of RefGen Tp_v2.0. We also found 1843 long non-coding RNAs, 1625 fusion genes, and 34,612 alternatively spliced events, with some transcript isoforms validated experimentally. A total of 16,734 differentially expressed genes were identified in the four tissues, including 43 isoflavonoid-biosynthesis-related genes, such as stem-specific expressed *TpPAL*, *TpC4H*, and *Tp4CL* and root-specific expressed *TpCHS*, *TpCHI1*, and *TpIFS*. Further, weighted gene co-expression network analysis and a targeted compound assay were combined to investigate the association between the isoflavonoid content and the transcription factors expression in the four tissues. Twelve transcription factors were identified as key genes for isoflavonoid biosynthesis. Among these transcription factors, the overexpression of *TpMYB30* or *TpRSM1-2* significantly increased the isoflavonoid content in tobacco. In particular, the glycitin was increased by 50–100 times in the plants overexpressing *TpRSM1-2*, in comparison to that in the WT plants. Our study provides a comprehensive and accurate annotation of the red clover transcriptome and candidate genes to improve isoflavonoid biosynthesis and accelerate research into molecular breeding in red clover or other crops.

## 1. Introduction

Red clover (*Trifolium pratense* L., Fabaceae) is a short-lived perennial forage plant that is cultivated for hay, grown in pastures to feed grazing livestock, and sown as a companion crop. It is adapted to a wide range of soil types and environmental conditions and yields productively in areas that are not suitable for growing alfalfa due to excessive soil acidity or soil moisture [1]. Red clover has a relatively high nitrogen-fixing ability and is nutritious for ruminant species. It is also known for its high levels of isoflavonoids. Although soybean is known as a rich source of isoflavones, red clover is comparatively more abundant (ca. 2 to 10-fold higher than that in soybean seeds) in formononetin and biochanin A, in particular. Isoflavonoids exhibit estrogenic activity and represent the most common group of natural phytoestrogens used in clinical nutrition. Isoflavone compounds are linked to the moderation of menopausal symptoms and to the prevention of prostate cancer [2]. They also show antioxidant activity and may act to protect the cardiovascular system [3].

Derived from the phenylpropanoid pathway, flavonoids are important secondary metabolites that include flavonols, anthocyanins, phlobaphenes, isoflavonoids, and condensed tannins (i.e., proanthocyanidins, PAs). Isoflavonoid biosynthesis is common in legumes; the synthesis starts with the deamination of phenylalanine into cinnamate by phenylalanine ammonia-lyase (PAL). Chalcone synthase (CHS) is a critical branch-node enzyme that generates naringenin chalcone or isoliquiritigenin chalcone, which act as flavonoid skeletons. Isoflavonoids are produced via a unique aryl migration reaction involving isoflavone synthase (IFS). The 2-hydroxyisoflavanone dehydratase (2HID) converts the isoflavonoid skeleton into genistein, daidzein, and glycitein [4]. Isoflavonoid biosynthesis involves an intricate network that includes many competing branch pathways. Previous studies have shown that altering the expression level of some structural genes, such as *CHS*, in the isoflavonoids synthesis pathway via genetic engineering cannot significantly increase the content of isoflavonoids [5]. Thus, recent studies on the regulation of flavonoid pathways have focused mainly on the characterization of transcription factors (TFs) that regulate key structural genes. In *Arabidopsis thaliana*, early structural genes in flavonoid biosynthesis—including *CHS, CHI1 (Chalcone-flavanone isomerase 1)*, and *F3H (Flavanone 3-hydroxylase)*—are positively regulated by three functionally redundant R2R3 MYB TFs, including AtMYB11, AtMYB12, and AtMYB111 [6,7]. The majority of R2R3 MYB TFs depend on a constitutive bHLH partner to activate target genes. To date, most of the structural genes in the flavonoid biosynthetic pathway have been coordinately regulated by a ternary complex comprising of MYB, bHLH, and WD-repeat proteins (MBW) among the plant species that have been studied. A similar complex involving TT2, TT8, and TTG1 has been demonstrated to play an important role in regulating proanthocyanidin accumulation in *Arabidopsis* [8]. Moreover, two other MBW regulatory complexes, ZmC1/ZmB/ZmPAC and PhAN2/PhAN1/PhAN11, have also been characterized in maize and petunia, respectively [9,10]. In *Arabidopsis*, the R3-MYB proteins such as AtMYBL2 negatively regulate flavonoid biosynthesis by directly interacting with the bHLH proteins to prevent R2R3 MYB TFs from binding to their bHLH partners and to inhibit the activity of the MBW complex [11]. However, little is known about the transcriptional regulation of the isoflavonoid biosynthesis pathway in red clover.

Red clover is an allogamous diploid species (2n = 2X = 14). The genome of red clover was first assembled from a genotype (cultivar “Milvus B”) using a protocol that integrated short-read next-generation sequencing (NGS) data, bacterial artificial chromosome (BAC) end sequences, one physical map, and two genetic maps [12]. However, in short-read RNAseq, it is difficult or even impossible to accurately predict each isoform. In contrast, single-molecule sequencing technologies (SMRT) such as the Pacific BioSciences (PacBio) sequencing platform enable much longer read lengths to be generated and thus provide a considerable advantage in the identification of transcriptome-wide full-length (FL) splice isoforms. Recently, SMRT has been used to characterize the complex transcriptomes of maize (*Zea mays*) [13], sorghum (*Sorghum bicolor*) [14], and cotton (*Gossypium barbadense*) [15].

Here, we conducted a new transcriptomics study for *T. pratense* using PacBio Iso-Seq (isoform sequencing) in four pooled tissue types, i.e., root, stem, leaf, and flower. In parallel, we conducted Illumina paired-end short-RNA-read sequencing, from the four tissue types (unpooled). Our results provide a more complete FL transcriptome that considerably enhances our knowledge of the red clover transcriptome. We also used the FL transcriptome to identify and characterize novel transcripts, AS events, associated splice isoforms, fusion transcripts, and long non-coding RNAs (lncRNAs). The identification of these genetic elements will improve the annotation of the *T. pretense* genome and enhance our understanding of transcription-level regulation of important traits in red clover. Moreover, we identified 12 differentially expressed bHLH and MYB transcription factors that may regulate the synthesis of isoflavonoids, and we verified the function of two MYBs (*TpMYB30* and *TpRSM1-2*) in the production of isoflavonoids in tobacco. This provides potential gene resources for genetic improvement of the synthesis of isoflavonoids in non-legume plants.

## 2. Results and Discussion

### 2.1. Red Clover Transcriptome Sequencing

To acquire accurate full-length RNA transcripts, a single library (0.5–6 kb) was constructed and sequenced using the PacBio Sequel platform with two SMRT cells for mixed equal amounts of total RNA from the root, stem, leaf, and flower tissue samples. This resulted in a total of 1118,821 raw polymerase reads with an average of 559,411 reads per cell (Table S1), as shown in Figure S1. After quality control, 751,364 high-quality reads of inserts (ROIs) were obtained (Table S1). Of these ROIs, 658,998 (87.7%) were classified as full-length transcripts and 92,365 (12.3%) as non-full-length transcripts, based on the presence and absence of 5′ primers, 3′ primers, and polyA tails, respectively (Table S1). The proportion of red clover ROIs accounting for FL transcripts was higher than the proportion reported in maize (43%) [13], switchgrass (28%) [16], and cotton (43%) [15]. Short (length < 300 bp) and chimeric reads (37,924) were discarded. A total of 621,074 full-length non-chimeric (FLNC) reads, with a mean length of 1822 bp, were kept for the subsequent analysis (Table S1).

To generate high-quality nonredundant isoforms, FLNC reads were subjected to Intelligent Clustering Engine (ICE) clustering to improve consensus accuracy and to generate polished full-length consensus sequences. In total, we obtained 273,494 consensus sequences, including 57,199 and 216,295 polished high-quality and low-quality consensus sequences, respectively. Error correction of these consensus sequences was performed using Illumina sequencing data to improve accuracy when mapping onto the red clover reference genome (Tp_v2.0). Mapping results included: (1) 1625 transcripts (0.6% of the total number of consensus sequences) that each mapped to at least two distinct genomic loci; (2) 271,591 transcripts (96.9%) that each mapped to a single, unique genomic locus; (3) 7092 transcripts (2.5%) that each showed no significant match to any genomic locations. After further filtering of isoforms with at least 90% alignment identity and 85% sequence coverage, 202,990 nonredundant consensus sequences were collapsed into 50,922 nonredundant consensus isoforms. 

**Figure 1:**
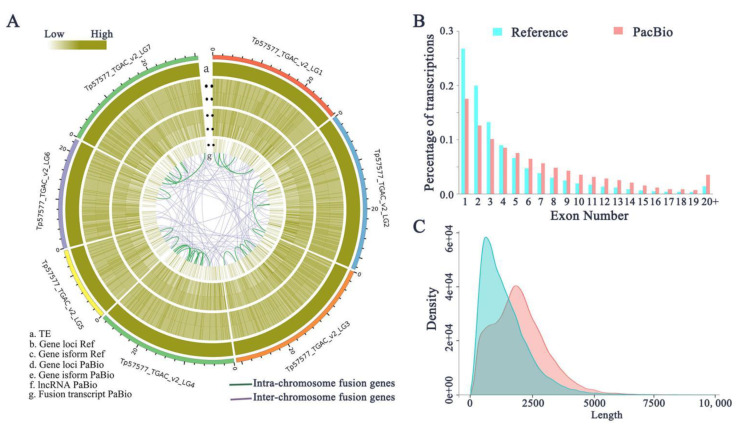
Comparison of the isoform annotations of the red clover v2.0 (reference) and PacBio Iso-Seq dataset. (**A**) Distribution of genomic features within the PacBio isoform dataset against the Tp_v2.0 genome assembly. (**B**) Distribution of the percentage of transcripts with different exon numbers in the reference genome and in the PacBio Iso-Seq dataset. (**C**) Comparison of isoform length in the gene model and PacBio Iso-seq dataset.

Further, we compared the loci coverage of the PacBio isoform dataset against the Tp_v2.0 genome assembly, which contains 41,302 transcripts covering 39,948 loci. The gene density of the PacBio dataset was lower than that of the Tp_v2.0 genome assembly, although it was higher in some specific regions (Figure 1A). A total of 50,922 isoforms covered 38,443 loci, where 8932 (17.5%) were single-exon isoforms and 41,990 (82.5%) were multiple-exon isoforms (Figure 1B). In contrast, in the reference annotation, 10,697 (26.8%) were single-exon isoforms and 29,210 (73.2%) were multiple-exon isoforms (Figure 1B). A total of 48,105 isoforms from the PacBio dataset were mapped to 19,860 known genes (representing 49.7% of the 39,948 genes). However, 2817 isoforms did not overlap with any existing annotated genes and therefore were identified as novel isoforms. We performed GO and KEGG functional enrichment analysis of all the novel isoforms, thus supplementing the annotation information of the Tp_v2.0 reference genome (Figure S2). The high percentage of new isoforms identified here demonstrates that PacBio full-length sequencing provides a more comprehensive set of isoforms than NGS-based methods [14].

In the Tp_v2.0 annotation, 1138 genes were annotated with two or more isoforms. For example, the locus with five isoforms was in the gene region of Tp_v2.0_scaf_1548: 11472–14327 (Tp_v2.0_gene39688.v2). In our PacBio dataset, 11,231 genes were identified with two or more isoforms. This dataset also showed significantly more isoforms per gene (mean = 3.5) than the Tp_v2.0 annotation (mean = 2.6), and the locus with the largest number of isoforms (nine) was Tp_v2.0_scaf_1033: 14887–17485 (Tp_v2.0_gene28536.v2). Thus, the new assembly of the transcriptome has a higher density of isoforms than that in the Tp_v2.0 assembly at the whole-genome level (Figure 1C), and may offer a better understanding of the real complexity of the transcriptome in red clover. 

### 2.2. Identification of Alternative Splicing Events and Fusion Transcripts

**Figure 2:**
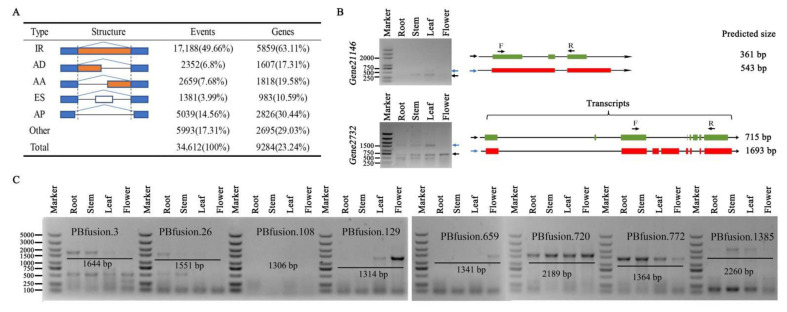
Characterization of AS events and validation of AS events and fusion transcripts using RT-PCR. (**A**) Classification of AS events. (**B**) RT-PCR validation of AS events in two genes. The transcripts structure of each isoform, from the Tp_v2.0 genome and PacBio dataset, are shown in green and red in the right panel, respectively. Green and red boxes show exons and lines with arrows show introns. PCR primers (F, forward and R, reverse) are shown on the first isoform of each gene. The length of each full-length isoform is shown behind the transcript structure. (**C**) RT-PCR validation of fusion transcripts.

Five major alternative splicing (AS) events were inferred: intron retention (IR), alternative 3′ splice sites (AA), alternative 5′ splice sites (AD), exon skipping (ES), and alternative position (AP) events. A total of 34,612 AS events were detected in 9284 genes using the Iso-Seq dataset (Table S2). Figure 2A shows the descriptive statistics of different AS events in red clover. In higher plants, AS plays a key regulatory role in modulating gene expression during plant development or in response to biotic and abiotic stresses [17]. We found that 63.11% of genes undergoing AS events were associated with IR events. AP events were the second most common type of AS event, followed by AA events. The number of AD and ES events were similar in red clover; both were consistent with previously published data [18]. 

Some genes exhibited more isoforms than previously annotated. For instance, 11 isoforms of gene1687 (encoding an MYB-family transcription factor) were found, but only two transcripts were annotated in the reference genome. We randomly selected six genes to validate the accuracy of the AS events analysis using a reverse transcription polymerase chain reaction (RT-PCR); the designed primers that could amplify all predicted transcripts are listed in Table S3. The sizes of amplified fragments were consistent with those of predicted fragments and the expression of some transcript isoforms exhibited tissue-specific patterns (Figure 2B and Figure S3). For example, a smaller transcript of gene21146 (encoding a photosystem I reaction center subunit N), which was produced by an IR event, was preferentially expressed in stem and leaf tissue in red clover (Figure 2B). The largest transcript of gene2732 (encoding a viral movement protein), which was produced by an ES event, was found to be expressed in root, stem, and leaf tissue, but not in flower tissue (Figure 2B).

Fusion transcripts result from trans-splicing events that join two separately encoded pre-RNAs into a single transcript. Fusion transcripts have been identified in diverse plant species [13,19], but the function was less characterized. Here, we identified 1625 full-length transcripts that mapped to two or more loci in the genome and were thus considered as fusion transcripts, and this analysis was supported by 1903 reads (Table S4). Fusion events were more likely to occur between chromosomes (1556) than within a chromosome (69) and tended to occur near chromosome termini (Figure 1A); similar results were also observed in other studies [13]. Eight candidate fusion transcripts were randomly selected to validate their authenticity using RT-PCR and Sanger sequencing. Seven of these fusion transcripts were confirmed (Figure 2C). Gene ontology analysis of fusion transcripts revealed that the majority were associated with catalytic activity and binding (category of molecular functions), metabolic/cellular processes (biological processes), and cells and membranes (cellular components) (Figure S4).

### 2.3. Long Non-Coding RNA Identification

**Figure 3:**
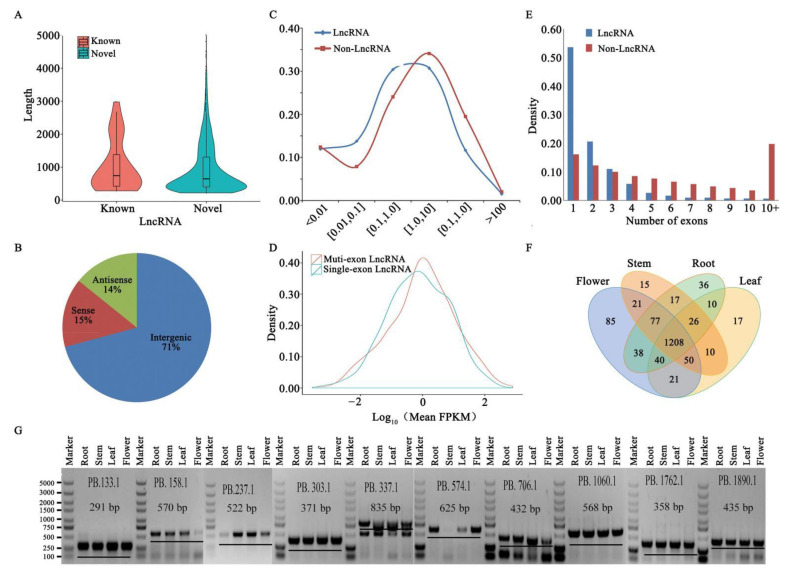
Characterization of identified lncRNAs. (**A**) Comparison of lengths of previously reported lncRNAs with novel lncRNAs identified in our study. (**B**) Proportions of three kinds of lncRNAs, classified according to their position relative to protein-coding genes. (**C**) Comparison of overall expression between lncRNAs and non-lncRNAs. (**D**) Comparison of overall expression between single-exon lncRNAs and multi-exon lncRNAs. (**E**) Number of exons in lncRNAs and non-lncRNAs. (**F**) Overlap of lncRNAs among the four tissue samples: root, stem, leaf, and flower. (**G**) Validation of 10 LncRNA transcripts using RT-PCR.

Long non-coding RNAs (lncRNAs) are defined as having length > 200 nt and are often species-specific. Recent studies supported the idea that lncRNAs play regulatory roles in numerous biological processes in plants [20]. In this study, 1843 lncRNAs were identified from 50,922 isoforms (Table S5). The lengths of lncRNAs ranged from 202 to 5012 bp, with a mean length of 944.3 bp, which is much shorter than the mean length of isoforms (i.e., 2008.47 bp) (Figure 3A). Mapping lncRNAs to the Tp_v2.0 chromosomes indicated that they showed a similar distribution to protein-coding genes, mostly located outside the pericentromeric regions (Figure 1A). Based on their position related to Tp_v2.0 annotations, we classified the lncRNAs into three groups: 70.8% were generated from intergenic regions, 14.2% from the antisense strand and 15.0% from the sense strand (Figure 3B). A comparison of overall expression between lncRNAs and non-lncRNAs showed that lncRNAs were expressed significantly less than non-lncRNAs (*p* < 0.01, Figure 3C) and multi-exon lncRNAs were expressed at a higher level than single-exon lncRNAs (Figure 3D), accounting for 51.6% of the total lncRNAs (Figure 3E). Among 1843 lncRNAs, 1208 were expressed in all four tissues, while other lncRNAs displayed tissue-specific expression patterns. For example, 85 lncRNAs were specifically expressed in flowers and 17 lncRNAs in leaves (Figure 3F). To further validate the lncRNAs, we randomly chose ten candidates, all of which were experimentally validated by RT-PCR (Figure 3G).

### 2.4. Analysis of Isoflavonoids and Expression Patterns of Isoflavonoid Biosynthetic Genes in Tissues

**Figure 4:**
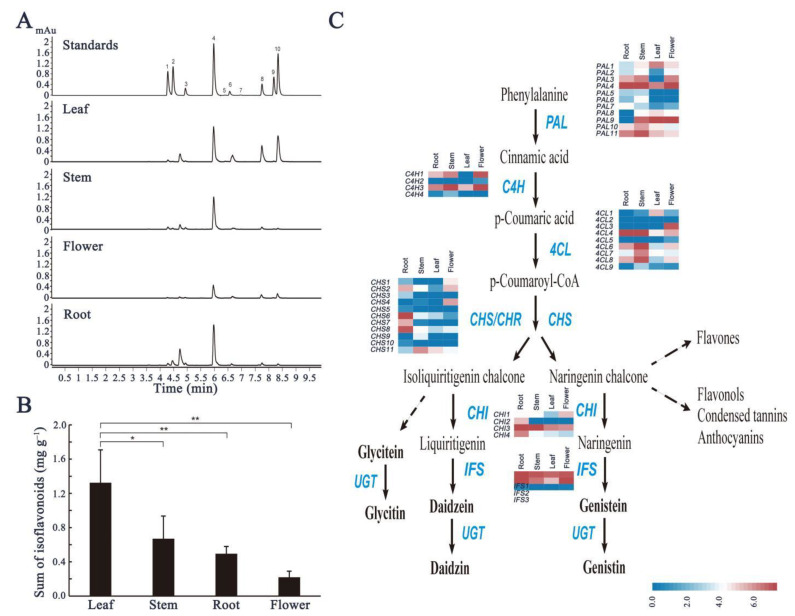
Content of selected isoflavonoids in different tissues of red clover and analysis of the differential expression of isoflavonoid synthesis structural genes. (**A**) HPLC/MS elution profiles of isoflavonoid components extracted from different tissues. Peak numbers corresponding to isoflavonoids are shown in Table S1. (**B**) Total isoflavonoid content (mg/g dry weight) in different tissues. Bar data are the means of three biological replicates ± SD, and ANOVA analyses were conducted using a one-way analysis of variance and Student’s *t*-test. Asterisks indicate a significant difference (* *p* < 0.05, ** *p* < 0.01). (**C**) Pathway of isoflavonoids biosynthesis in plants and the expression patterns of differentially expressed structural genes in four tissues, shown in heatmaps. *PAL*: phenylalanine ammonia-lyase; *C4H*: cinnamate-4-hydroxylase; *4CL*: 4-coumarate-CoA ligase; *CHS*: chalcone synthase; *CHR*: chalcone reductase; *CHI1*: chalcone isomerase; *IFS*: 2-hydroxyisoflavanone synthase; *UGT*: uridine diphosphate glycosyltransferase.

Isoflavonoids in plants are variable in quantity and are influenced by genetic and environmental factors. Additionally, they also vary among tissues and developmental stages [21]. We analyzed ten selected isoflavonoid components (daidzin, glycitein, genistein, ononin, daidzein, glycetein, genistin, formononetin, prunetin, and biochanin A) in samples of different tissues of red clover plants. Different isoflavonoids were identified by retention time, chromatographic behavior, and mass spectrometry using standards (Table S6), with previously published elution profiles [22]. We found that ononin was the most common individual isoflavonoid found in all four tissues, although the other nine were also identified. The distribution of each of these isoflavonoids also differed between leaf and root tissues. In the leaf, ononin, glycetein, and formononetin predominated in the isoflavonoids profile, while genistin and ononin predominated in root tissue (Figure 4A; Table S7). The total isoflavonoid content of the four tissues showed significant differences, with the highest in leaf samples (with a mean total isoflavonoid content of 1.324 mg g^−1^ DM (dry mass)), followed by stem (mean = 0.669 mg g^−1^ DM), root (mean = 0.494 mg g^−1^ DM), and flower (mean = 0.220 mg g^−1^ DM) samples (Figure 4B; Table S7).

Isoflavonoid biosynthesis is catalyzed by multiple enzymes that are encoded by structural genes via a legume-specific branch of the phenylpropanoid pathway (Figure 4C), which produces a variety of specialized metabolites, including flavonoids, anthocyanins, stilbenoids, lignin, and isoflavonoids. In this study, we identified 16,734 differentially expressed genes in different tissues of red clover. We further analyzed the relative FPKM of 42 structural genes in isoflavonoid biosynthesis, identified by a functional enrichment analysis (Figure 4C; Table S8). Eleven TpPAL genes exhibited different expression patterns in tissues, such as TpPAL1, TpPAL8, and TpPAL9, mainly expressed in the leaf, while the expression of TpPAL7 was highest in the root, and expression of other TpPALs were highest in the stem (Figure 4C). Relative expressions of most TpPALs, TpC4Hs, and Tp4CLs were at high levels in the stem, indicating their possible role in lignin synthesis to improve the mechanical strength and compressive capacity of the stem [23]. In addition, TpCHSs, TpCHIs, and TpIFSs, were most strongly expressed in the root (Figure 4C). Clearly, this does not explain the fact that isoflavonoids mainly accumulate in leaves. These results indicate that isoflavonoids may possibly be synthesized in large quantities in the roots, but are mainly transported to, and stored in, the leaves. Similarly, a high concentration of isoflavonoids has been found in mature soybean seeds and leaves, but the highest expression level of IFS1 was observed in the root and seed coat [24]. This suggests that transport of isoflavonoids between different organs occurs within the plant. Some natural products in plants are often transported from the site of synthesis to the site of accumulation [25].

### 2.5. Co-Expression Network Analysis of Transcription Factors by WGCNA

**Figure 5:**
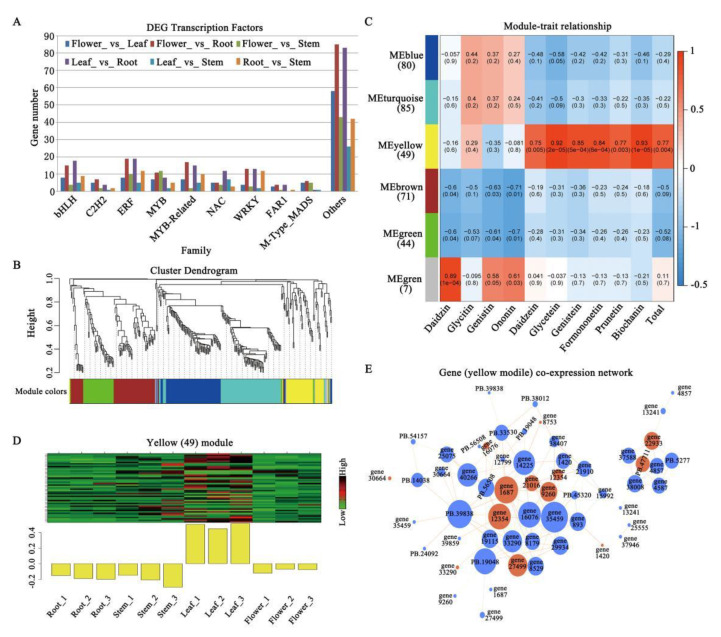
The WGCNA of differentially expressed transcription factors in four tissues (root, stem, leaf, and flower). (**A**) Differentially expressed TF families of genes. (**B**) The cluster dendrogram of differentially expressed transcription factors. Each branch in the figure represents one gene, and every color below represents one co-expression module. (**C**) Heatmap of the correlation between module and the content of isoflavonoids. The color of each cell at the row–column intersection indicates the correlation coefficient between the module and isoflavonoid content. The color scale indicates the magnitude of the correlation coefficient, ranging from low (blue) to high (red). (**D**) Heatmap and expression pattern of the genes in different tissues in the yellow module. (**E**) Visualization of connections of genes in the yellow module. Each node represents a gene and the connecting lines (edges) between genes represent co-expression correlations (Supplementary Table S9). Forty-three TFs with edge weights ≥ 0.2 are visualized using Cytoscape. Thirty-two TFs are shown by larger circles. Twelve hub genes belonging to the bHLH, MYB, and MYB-related TF families are shown in red.

Spatiotemporal transcriptional regulation of metabolic pathways is controlled by a complex network of transcription factors (TFs) [26]. Here, we investigated the expression of TFs in different tissues in red clover. Among the 2071 TFs detected, 336 TFs were found to be differentially expressed among the four tissues. We classified the TFs into 41 families, including bHLH, MYB, and MYB-related families (Figure 5A). MYB, bHLH, and WD-repeat proteins have been known to form a transcriptional complex, which regulates the expression of structural genes in the flavonoid biosynthesis pathway [27]. 

To identify the TFs related to isoflavonoid biosynthesis, we conducted a weighted gene co-expression network analysis (WGCNA) using all the 336 differentially expressed TFs. These TFs were divided into five distinct modules, within which the genes were highly correlated (labeled by different colors) (Figure 5B; Table S9). An analysis of module trait relationships revealed that the “yellow” module was highly correlated with a content of six isoflavonoids (Figure 5C). In particular, the yellow module was significantly correlated with the content of glycetein (r= 0.92, *p* = 2 × 10^−5^) and biochanin A (r = 0.93, *p* = 1 × 10^−5^) in leaves (Figure 5C,D). This is consistent with the accumulation of isoflavonoids in leaves (Figure 4B).

We constructed a co-expression network to identify hub genes from the yellow module (Table S10), i.e., highly connected TFs, which may play a central role in isoflavonoid biosynthesis. Remarkably, 12 of the 43 hub genes were found to belong to the bHLH, MYB, and MYB-related families, including five TpbHLH genes (gene22933, gene27499, gene30664, gene33290, and gene8753), two TpMYBs (PB47711 and gene9260), and five TpMYB-related genes (gene12354, gene1420, gene16076, gene1687, and gene21016), which are marked as red circles in the co-expression network (Figure 5E). GmMYB29 was reported to regulate isoflavonoid biosynthesis in soybean by trans-activating the GmIFS2 (isoflavone synthase 2) and GmCHS8 (chalcone synthase 8) gene promoters [5]; GmMYB58 and GmMYB205 are seed-specific activators for isoflavonoid biosynthesis [28]; GmMYB176 regulates GmCHS8 gene expression and then affects isoflavonoid biosynthesis [29]. In Lotus japonicus, LjMYB14 has been found to be constitutively overexpressed, and it induces the expression of at least 12 structural genes in the phenylpropanoid and isoflavonoid pathways [30]. However, whether the hub genes participate in the transcriptional regulation of isoflavonoid synthesis or play a positive or negative role in expression of the structural genes, requires further investigation.

### 2.6. TpMYB30 and TpRSM1/2 Increased the Content of Isoflavonoids in Tobacco

In this study, an MYB TF (gene9260) and two MYB-related genes (gene1420 and gene16076) were selected for further characterization of the relation to their counterparts in other species. Through a phylogenetic analysis, we found that gene9260 was closest to AtMYB30 (Figure S5), which was denoted TpMYB30. The BLAST results showed that the amino sequences of gene1420 and gene16076 have 79% and 78% similarity, respectively, in query coverage and 66% and 65% identity with AtRSM1 (RADIALIS-LIKE SANT/MYB1). We thus denoted gene1420 and gene16076 as TpRSM1-1 and TpRSM1-2, respectively.

**Figure 6:**
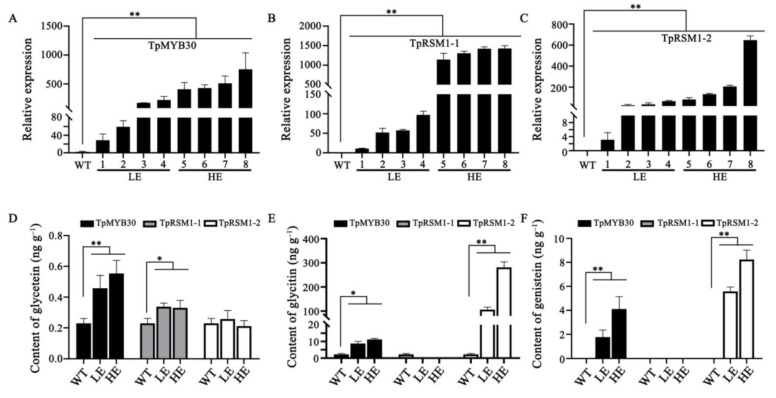
Effect of three MYB genes (*TpMYB30*, *TpRSM1-1,* and *TpRSM1-2*) on isoflavonoids biosynthesis. (**A**) The expression of *TpMYB30* in wild-type and transgenic plants overexpressing *TpMYB30*. (**B**) The expression of *TpRSM1-1* in wild-type and transgenic plants overexpressing *TpRSM1-1*. (**C**) The expression of *TpRSM1-2* in wild-type and transgenic plants overexpressing *TpRSM1-2*. (**D**) The content of glycetein in wild-type and transgenic plants overexpressing *TpMYB30*, *TpRSM1-1*, and *TpRSM1-2*, respectively. (**E**) The content of glycitin in wild-type and transgenic plants overexpressing *TpMYB30*, *TpRSM1-1,* and *TpRSM1-2*, respectively. (**F**) The content of genistein in wild-type and transgenic plants overexpressing *TpMYB30*, *TpRSM1-1,* and *TpRSM1-2*, respectively. Bar data are the means of three biological replicates ± SD, and ANOVA analyses were conducted using one-way analysis of variance and Student’s *t*-test. Asterisks indicate significant difference (* *p* < 0.05, ** *p* < 0.01). The samples overexpressing *TpMYB30*, *TpRSM1-1,* and *TpRSM1-2* were divided into two groups: relatively low-expression (LE) and high-expression (HE) groups.

Tobacco (*Nicotiana tabacum*) and Arabidopsis thaliana were considered not to produce isoflavonoids in previous studies, since there is no isoflavone synthase (IFS) in the two species. Additionally, isoflavonoids were detected after expressing GmIFS in tobacco [31]. However, some studies showed that isoflavonoids were naturally present in tobacco leaves and Arabidopsis, using HPLC-MS methods. For instance, Lapcik et al. [32] found that isoflavonoids were present in Arabidopsis. Additionally, isoflavonoids were also found in leaves and flowers of several Nicotiana species [33,34]. We investigated the potential function of TpMYB30, TpRSM1-1, and TpRSM1-2 in the biosynthesis of isoflavonoids by overexpressing the three genes in tobacco via Agrobacterium-mediated transformation. The expression of all the three genes in the transgenic lines was significantly higher than that in the wild type. The transgenic lines were divided into LE (relatively low-expression) and HE (high-expression) groups for the detection of the presence of isoflavonoids (Figure 6A–C). We compared the content of isoflavonoids (glycetein, glycitin, and genistein) in leaves of transgenic tobacco plants and wild-type tobacco (WT). In the WT plants, the contents of glycetein and glycitin were 0.230 ng g^−1^ and 2.184 ng g^−1^, respectively, but genistein was not detected. In contrast, glycetein (0.402–0.650 ng g^−1^), glycitin (7.298–11.694 ng g^−1^), and genistein (1.131–5.106 ng g^−1^) (Figure 6D–F and Table S11) were found in the TpMYB30 transgenic lines. In TpRSM1-2 lines, glycitin and genistein were significantly increased compared to the WT (Figure 6D–F), but glycetein showed no difference compared to the WT (Figure 6D). In particular, glycitin was increased up to 106.35–281.38 ng g^−1^ in the TpRSM1-2 lines, which was 50–100 times higher than in WT plants (Figure 6E and Table S11). Genistein reached 5.277–8.971 ng g^−1^ in the TpRSM1-2 lines (Figure 6F and Table S11). However, the glycetein in the TpRSM1-1 lines was slightly higher than in the WT plants, and glycitin and genistein were not detected (Figure 6D,E). These results strongly suggest that TpMYB30 and TpRSM1-2 may be important regulators for isoflavonoid biosynthesis in red clover, but not TpRSM1-1, for which the isoflavonoids are at very low levels or below detection.

The overexpression of TpMYB30 and TpRSM1-2 also significantly increased the content of other flavonols. Compared with the WTs, four flavonoids (naringenin, dihydrokaempferol, kaempferide, and epicatechin) and two flavones (apigenin and luteolin-7-O-glucoside) were increased in the TpMYB30 lines (Table S11). In the TpRSM1-2 plants, two phenolic compounds (p-coumaric acid and ferulic acid), three flavonoids (naringenin, epicatechin, and epigallocatechin) and four flavones (apigenin, luteolin-7-O-glucoside, quercitrin, and rutin) were higher than in the WT. Nevertheless, phenylalanine, which is the initial substrate in the phenylpropanoid biosynthesis pathway, was significantly decreased in the TpRSM1-2 lines (Table S11). Interestingly, we found that the expression of TpRSM1-2 was positively correlated with the expression of three TpPAL homolog genes (Figure S6). Therefore, we infer that TpRSM1-2 may positively regulate the expression of TpPAL genes, which utilize phenylalanine for the biosynthesis of the downstream metabolites, including isoflavonoids. 

Multiple health-promoting effects of isoflavonoids, such as the use of genistein and daidzein against hormone-related cancers, osteoporosis, menopausal symptoms, and cardiovascular disease, have been reported [35]. Epidemiological studies showed that a high consumption of soybean-derived foods was associated with a low incidence of diseases, and the health-protective activities could be ascribed to isoflavonoids [36]. Therefore, metabolic engineering of isoflavonoids in more widely consumed non-legume plants (vegetables, grains, and fruits) has attracted great interest, to enhance the dietary intake of these compounds. In our study, we found that two novel TFs (TpMYB30 and TpRSM1-2) positively regulate the biosynthesis of genistein, glycetein, and glycitin in tobacco, especially, the overexpression of TpRSM1-2, which leads to a great increase in glycitin.

## 3. Materials and Methods

### 3.1. Plant Materials

Red clover variety “ZW2780” was grown under natural conditions (i.e., the annual average sunshine of 2684 h, temperature of 11.8 °C, and precipitation of 550.3 mm) in Changping experimental station, Chinese Academy of Agricultural Science (Beijing, China; 40°18′ N, 116°24′ E). Root, stem, leaf, and flower samples were collected when flowers reached the full-bloom stage. Samples were immediately frozen in liquid nitrogen for subsequent RNA extraction. For each tissue, at least three plants were pooled, and three biological replicates were used for RNA extraction. Total RNA was extracted using RNeasy Plant Mini Kit (QIAGEN, Germany) as per the manufacturer’s instructions.

### 3.2. Sample Preparation and HPLC/MS Analysis

Isoflavonoids in four tissues (roots, stems, leaves, and flowers) of red clover were detected using HPLC/MS analysis at Shanghai Applied Protein Technology Co. Ltd. (Shanghai, China). Briefly, samples were dried at 65 °C for 48 h and ground into powder. Then, 0.025 g of sample was immersed in 1 mL of 80% (*v*/*v*) aqueous methanol and ultra-sonicated for 20 min (ultrasonic power 100 W, temperature 45 °C), then placed in a refrigerator at 4 °C for 12 h. Next, the supernatant extract was collected by centrifugation (13,000 rpm, 5 min, 4 °C) and separated using a ZORBAX Eclipse XDB-C18 column (100 mm × 2.1 mm, 1.8 µm). The column was attached to an Agilent 6470 triple quadrupole MS system to identify and quantify the various isoflavonoids present in samples by comparing their mass spectra with those of the standards. All solvents (water, methanol, and acetonitrile) were HPLC grade. The binary mobile phase consisted of water (solvent A) and acetonitrile (solvent B). A gradient elution program was employed with a flow rate of 0.3 mL/min as follows: 80% A from 0 to 1 min; 80% to 0% A from 1 to 10 min; 0% to 0% A from 10 to 11 min; 0% to 80% A from 11 to 11.1 min; hold 80% A from 11.1 to 13 min. The injection volume was 2 µL for all the standards and samples. Isoflavonoids were analyzed with an electrospray ionization source operated in positive ion mode. Instrument settings were as follows: dry gas temperature, 350 °C; dry gas flow, 8 L min^−1^; capillary voltage, 3500 V; nebulizer, 45 bar. Data were analyzed using a one-way analysis of variance and Student’s *t*-test, and *p*-values < 0.05 or 0.01 were considered to be significant. The standard deviations (SDs) were calculated using data from three biological replicates.

### 3.3. Library Preparation and PacBio Sequencing

We pooled equal amounts of red clover RNA from each of the four tissues prior to library construction for PacBio sequencing. RNAs were first reverse-transcribed using a SMARTer^®^ PCR cDNA Synthesis Kit. PCR amplification was carried out using KAPA HiFi PCR Kits. The product was separated by agarose-gel-based size selection into cDNA fractions of length 0.5–6 kb. The cDNA products were then purified for library construction using a SMRTbell Template Prep Kit 1.0. Two SMRTbell libraries were sequenced on PacBio Sequel long-read sequencers using V2 polymerase chemistry and 600 min movie times at Nextomic (Wuhan, China).

### 3.4. Illumina RNA Sequencing

An Illumina HiSeq X Ten platform was used to generate paired-end reads to correct PacBio reads and quantify splicing. Total RNA was extracted and evaluated as described above. Strand-specific RNA-seq libraries were constructed using 5 µg of total RNA from four tissues (replicated three times) and a dUTP strand-specific library protocol. Strand-specific libraries were sequenced as 125 nt paired-end reads at Nextomic. To obtain clean reads, we removed adapter sequences, poly-N reads, and low-quality reads from the raw data using an NGS QC Toolkit (version 2.3). High-quality reads were then mapped to the red clover RefGen Tp_v2.0 sequence using TopHat2 with the default parameters. Only reads with a perfect match or one mismatch were analyzed further and annotated based on the reference genome. Gene expression levels expressed as fragments per kilobase of transcript per million fragments (FPKM) were calculated using Cuffquant and Cuffnorm software. Differentially expressed genes were identified by DESeq with |log2 (foldchange)| ≥ 1 and *p* < 0.05 as the threshold. The *p*-values were adjusted using the Benjamini and Yekutieli approach for controlling false discovery rates [37].

The raw sequence data from both the PacBio and Illumina RNA sequencing have been deposited in the Genome Sequence Archive (CRA001471) in Beijing Institute of Genomics (BIG) Data Center, Chinese Academy of Sciences (http://bigd.big.ac.cn/gsa, accessed on 18 August 2021).

### 3.5. Functional Annotation of Transcripts

All known and newly predicted genes were annotated using public databases, including the NCBI nonredundant protein database (Nr), the NCBI nonredundant nucleotide database (Nt), and the Swiss-Prot, the Gene Ontology (GO), the Kyoto Encyclopedia of Genes and Genomes (KEGG), and the Clusters of Orthologous Groups (COG) databases. Searches were performed using the BLASTX algorithm with an E-value threshold of 10-5.

### 3.6. PacBio Iso-Seq Data Processing and Read Correction

PacBio data were processed and evaluated with several tools implemented by SMRT Link version 5.0 (https://github.com/PacificBiosciences/SMRT-Link/wiki/Support, accessed on 3 September 2017). Raw reads in fastq format were extracted from h5-formatted files by bash5tool in the pbh5tools package. The suggested parameters (i.e., MinReadScore = 0.75, MinSRL = 50, and MinRL = 50) were used to filter and trim raw reads. Next, clean polymerase reads were processed to separate reads of inserts with pass > 1 and accuracy > 0.8. Chimeras, artificial concatemers and fusion genes were removed using the SMRT Iso-Seq analysis pipeline (http://www.pacb.com/products-and-services/analytical-software/smrt-analysis/, accessed on 4 September 2017). Only full-length non-chimeric (FLNC) reads were kept for downstream analysis. Using the isoseq_cluster panel, we selected the ‘Predict Consensus Isoforms using the ICE Algorithm’ and ‘Call Quiver to Polish Consensus Isoforms’ options to obtain high-quality, full-length, polished consensus transcripts. LoRDEC software was used to correct the sequencing errors in consensus transcripts using Illumina reads as a reference [38]. The corrected consensus transcripts were then mapped by GMAP with > 85% alignment coverage and > 90% alignment identity [39]. Redundant isoforms were further removed using collapse_isoforms_by_sam.py, as implemented in pbtranscript-tofu (version 2.2.3).

### 3.7. Identification and Functional Annotation of Novel Transcripts

Full-length transcripts were aligned to gene models of the red clover genome [12]. Those that could not be aligned were considered to be novel transcripts. We used WEGO for GO enrichment analysis [40]. KEGG pathway mapping was performed using the KEGG Automatic Annotation Server (KAAS) version 2.0.

### 3.8. Identification of Alternative Splicing Events, Fusion Transcripts, and LncRNA

The alternative splicing (AS) events were identified from alignments using a Python script (alternative_splice.py, source: https://github.com/Nextomics/pipeline-for-isoseq, accessed on 20 September 2017). AS events were classified as exon skipping (ES), intron retention (IR), alternative donor site (AD), alternative acceptor site (AA), and alternative position (AP) events. A Python script (fusion_finder.py) in the pbtranscript-tofu package (http://github.com/PacificBiosciences/cDNA_primer/, accessed on 20 September 2017) was used to identify fusion transcripts. We used the following criteria to identify fusion transcripts: (a) FL transcripts were mapped to two or more loci in the reference genome; (b) each mapped locus must align with at least 10% of the transcript; (c) the combined alignment coverage must be at least 99%; (d) mapped loci must be at least 10 kb apart. To further exclude putative false candidates, transcripts involving two or more genes from the same gene family were discarded. Novel transcripts were processed to identify long non-coding RNAs using the lncRNAs pipeline (https://bitbucket.org/arrigonialberto/lncrnas-pipeline, accessed on 30 September 2017).

### 3.9. Construction and Visualization of Co-Expression Network

We performed a weighted gene co-expression network analysis using the WGCNA (version 1.66) R package [41]. The constructed network was visualized using Cytoscape version 3.6.1.

### 3.10. Transformation of Tobacco Plants and Analysis of Isoflavonoids in Leaves

The coding regions of a red clover MYB TF gene (TpMYB30) and two MYB-related genes (TpRSM1-1 and TpRSM1-2) were amplified and inserted into the pBI121 vector to generate overexpression constructs, followed by transformation into EHA105 for Agrobacterium-mediated transformation in tobacco plants. Transgenic plants were confirmed by PCR. The expression of the three genes in transgenic plants and a wild type was determined by quantitative real-time PCR (qRT-PCR), using the SYBR^®^ Premix Ex Taq™ II (Perfect Real Time) kit (Takara, Japan) with gene-specific primer pairs. The assay of isoflavonoids in the transgenic tobaccos was conducted by the same LC/MS method as described above. All data were analyzed using a one-way analysis of variance and Student’s *t*-test, and *p*-values < 0.05 or 0.01 were considered to be significant. The standard deviations (SDs) were calculated with data from three biological replicates.

## 4. Conclusions

In conclusion, we obtained full-length transcriptomes of *T. pretense*, which revealed complex transcripts such as alternatively spliced isoforms, long non-coding RNAs, fusion genes, and novel isoforms, thus providing more accurate annotation of the red clover genome. Our results facilitate the understanding of transcriptional complexity in plants. Isoflavonoid profiling and gene expression pattern analysis suggested that isoflavonoids may be synthesized in red clover roots and then transported to leaves. We identified key regulators of isoflavonoid synthesis, which include bHLH, MYB, and MYB-related TFs. We demonstrated that *TpMYB30* and *TpRSM1-2* were directly involved in isoflavonoid biosynthesis through transgenic experiments. This study provides important resources for the discovery of more genes to enhance our understanding of isoflavonoid biosynthesis in red clover and other legumes, and to enrich isoflavonoids in non-legume crops via genetic engineering.

## Data Availability

The raw sequence data have been deposited in the Genome Sequence Archive (CRA001471) in Beijing Institute of Genomics (BIG) Data Center, Chinese Academy of Sciences (http://bigd.big.ac.cn/gsa, accessed on 18 August 2021).

## Supplementary Materials

The following are available online at https://www.mdpi.com/article/10.3390/ijms222312625/s1.
