# Supplementary material for: Unveiling the Complexity of Red Clover (Trifolium pratense L.) Transcriptome and Transcriptional Regulation of Isoflavonoid Biosynthesis Using Integrated Long- and Short-Read RNAseq
Source: Int J Mol Sci. 2021 Nov 23;22(23):12625. doi: 10.3390/ijms222312625 (PMC8658037; doi:10.3390/ijms222312625)
Supplement: Supplementary file 1 [file ijms-22-12625-s001.zip › supplementary figures.pdf]

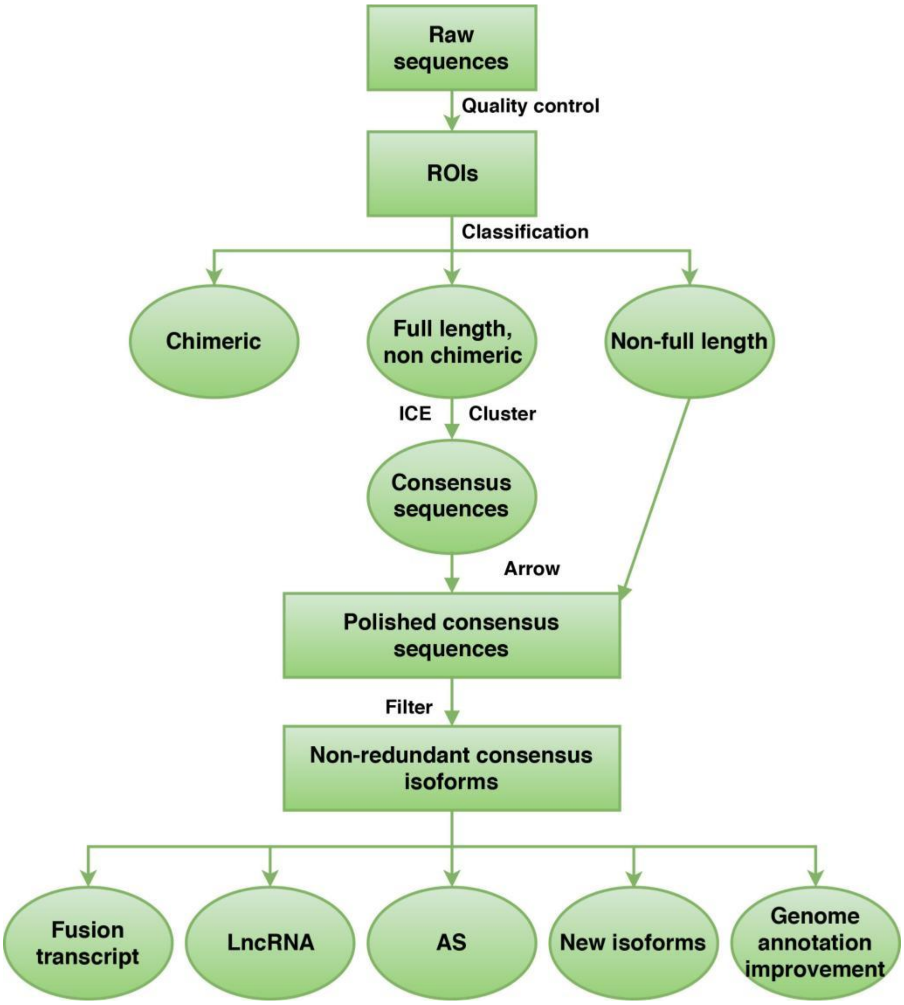

Figure S1. Iso-Seq workflow for data processing

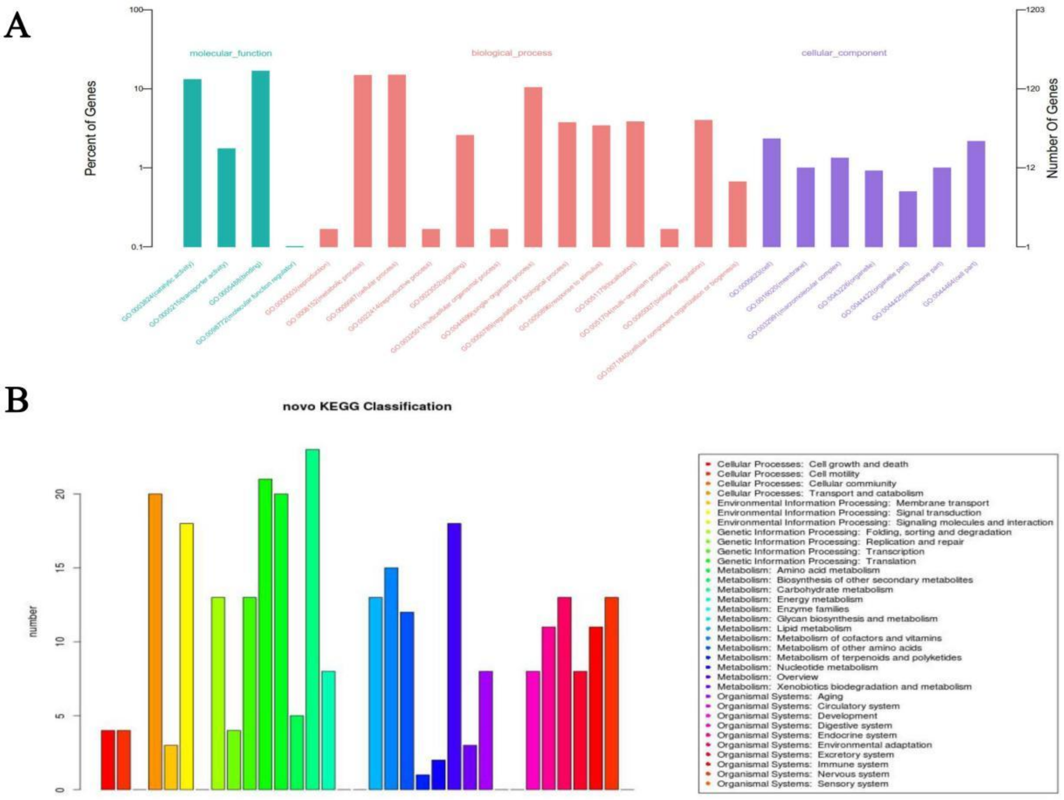

Figure S2. GO and KEGG enrichment annotation of novel isoforms

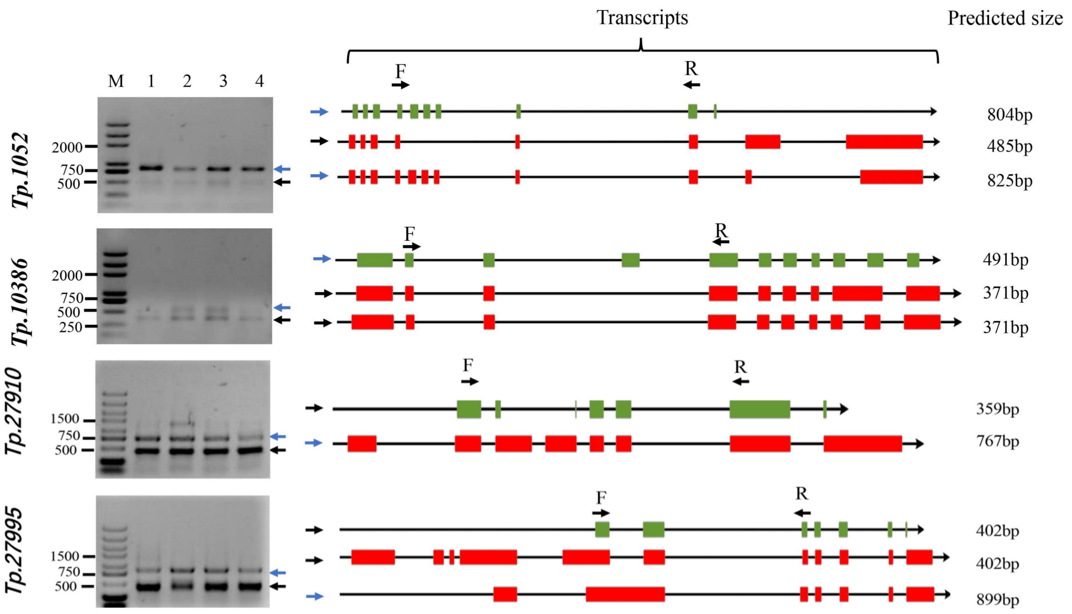

Figure S3. RT-PCR verification of alternative splicing events

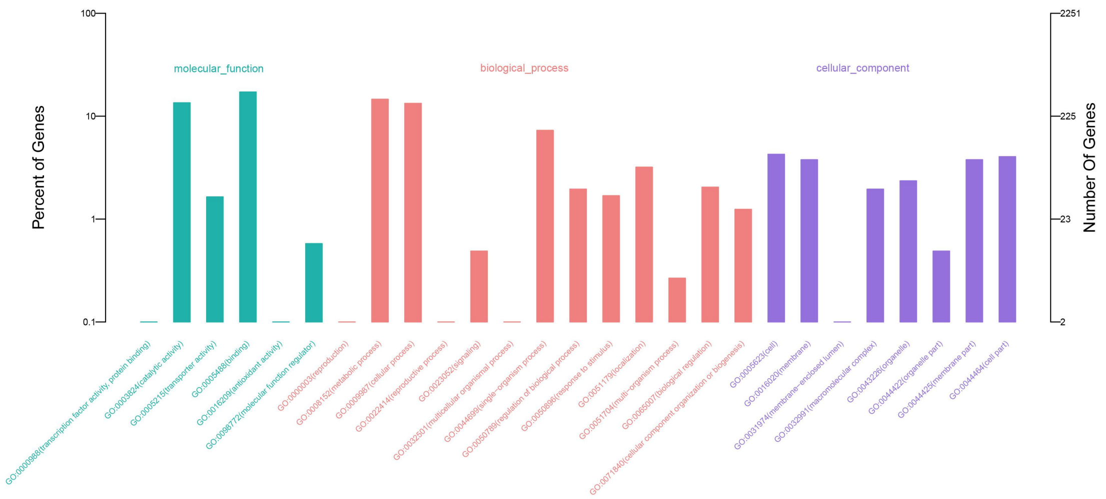

Figure S4. GO enrichment annotation of fusion transcripts

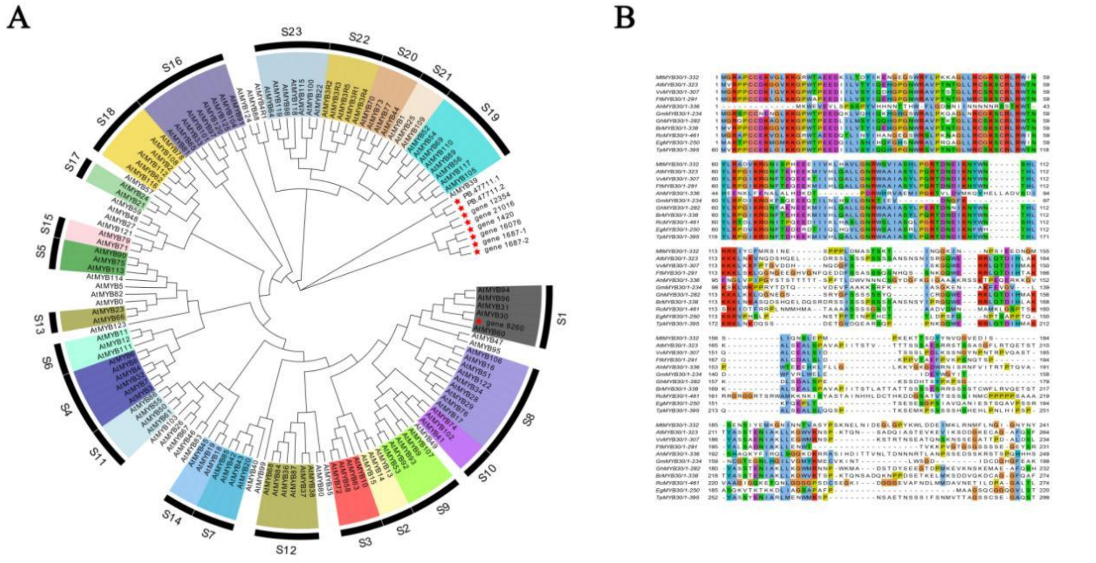

Figure S5. The phylogenetic tree analysis of some hub genes, including MYB and MYB-related genes and multiple sequence alignment of the TpMYB30 with some MYB30 from other species.

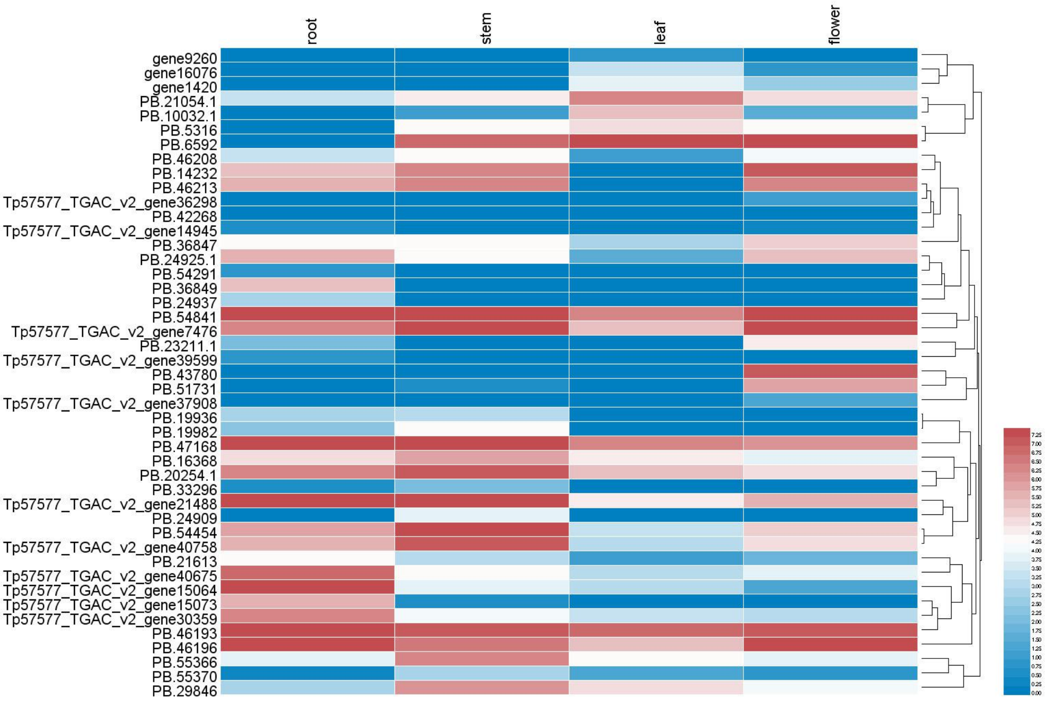

Figure S6. co-expression analysis between three hub genes (TpMYB30, TpRSM1-1 and TpRSM1-2) and the differential expressed structural genes involved in isoflavonoids biosynthesis.
